# Identification of a C/G polymorphism in the promoter region of the *BRCA1* gene and its use as a marker for rapid detection of promoter deletions

**DOI:** 10.1038/sj.bjc.6690122

**Published:** 1999-02

**Authors:** A Catteau, C-F Xu, M A Brown, S Hodgson, J Greenman, C G Mathew, A M Dunning, E Solomon

**Affiliations:** Division of Medical and Molecular Genetics,UMDS, 8th Floor, Guy's Tower, Guy's Hospital, London, SE1 9RT, UK; CRC Human Cancer Genetics Research Group, Box 238, Addenbrooke's Hospital, Hills Road, Cambridge, CB2 2QQ, UK

**Keywords:** breast cancer, *BRCA1* promoter, C/G polymorphism, linkage disequilibrium, deletion marker

## Abstract

Reduced expression of *BRCA1* has been implicated in sporadic breast cancer, although the mechanisms underlying this phenomenon remain unclear. To determine whether regulatory mutations could account for the reduced expression, we screened the promoter region by sequencing in 20 patients with sporadic disease. No mutations were detected; however, a new polymorphism consisting of a C-to-G base change within the β-promoter was identified, with the frequency of the G allele being 0.34. Close to complete linkage disequilibrium was found between this marker and the Pro871Leu polymorphism, situated in exon 11, which has previously been shown not to be associated with breast or ovarian cancer. This indicates that the C/G polymorphism is also unlikely to play a role in either disease. However, the strength of linkage disequilibrium between these markers permitted their use for rapid screening for genomic deletions within *BRCA1*. A series of 214 cases with familial breast cancer were analysed using this approach; 88/214 were heterozygous for the promoter polymorphism, thereby excluding a deletion in this region. Among the remaining patients, one hemizygous case reflecting a promoter deletion was successfully identified. Therefore, this study indicates that deletions within the β-promoter region of *BRCA1* are an uncommon event in familial breast cancer. Furthermore, it suggests that mutations within the *BRCA1* promoter are unlikely to account for the reported decreased expression of *BRCA1* in sporadic disease. *1999 Cancer Research Campaign*


*British Journal of Cancer* (1999) **79**(5/6), 759–763
© 1999 Cancer Research Campaign
Article no. bjoc.1998.0122

### Identification of a C/G polymorphism in the promoter region of the *BRCA1* gene and its use as a marker for rapid detection of promoter deletions

**A Catteau[1], C-F Xu[1], MA Brown[1], S Hodgson[1], J Greenman[1], CG Mathew[1], AM Dunning[2] and E Solomon[1]**

[1]Division of Medical and Molecular Genetics, UMDS, 8th Floor, Guy's Tower, Guy's Hospital, London SE1 9RT, UK; [2]CRC Human Cancer Genetics Research Group, Box 238, Addenbrooke's Hospital, Hills Road, Cambridge CB2 2QQ, UK

**Summary** Reduced expression of *BRCA1* has been implicated in sporadic breast cancer, although the mechanisms underlying this phenomenon remain unclear. To determine whether regulatory mutations could account for the reduced expression, we screened the promoter region by sequencing in 20 patients with sporadic disease. No mutations were detected; however, a new polymorphism consisting of a C-to-G base change within the β-promoter was identified, with the frequency of the G allele being 0.34. Close to complete linkage disequilibrium was found between this marker and the Pro871Leu polymorphism, situated in exon 11, which has previously been shown not to be associated with breast or ovarian cancer. This indicates that the C/G polymorphism is also unlikely to play a role in either disease. However, the strength of linkage disequilibrium between these markers permitted their use for rapid screening for genomic deletions within *BRCA1*. A series of 214 cases with familial breast cancer were analysed using this approach; 88/214 were heterozygous for the promoter polymorphism, thereby excluding a deletion in this region. Among the remaining patients, one hemizygous case reflecting a promoter deletion was successfully identified. Therefore, this study indicates that deletions within the β-promoter region of *BRCA1* are an uncommon event in familial breast cancer. Furthermore, it suggests that mutations within the *BRCA1* promoter are unlikely to account for the reported decreased expression of *BRCA1* in sporadic disease.

**Keywords:** breast cancer; *BRCA1* promoter; C/G polymorphism; linkage disequilibrium; deletion marker

The breast cancer susceptibility gene *BRCA1* was isolated in 1994 (Miki et al, 1994). Since then, more than 300 distinct disruptive germline mutations within the coding region of the gene have been identified in familial breast and ovarian cancer (Couch et al, 1996). However, no somatic *BRCA1* mutations have been found in sporadic breast cancer and they have been found only rarely in sporadic ovarian cancer (Futreal et al, 1994; Hosking et al, 1995; Merajver et al, 1995). Nevertheless, the high degree of loss of heterozygosity at the *BRCA1* locus and reduced *BRCA1* expression in sporadic breast tumours suggest that *BRCA1* may also play a role in tumorigenesis in sporadic disease (Thompson et al, 1995). The mechanisms accounting for reduced levels of *BRCA1* mRNA in this context have not been clearly established. This effect may be mediated through decreased transcription because of mutations within the promoter region or epigenetic mechanisms. We have previously examined the promoter region of the human *BRCA1* gene in detail, defined potential transcription factor binding sites and have demonstrated its complex regulation (Xu et al, 1995; Brown et al, 1996; Xu et al, 1997). The *BRCA1* gene has two promoters (α and β), controlling the expression of two distinct transcripts α and β; the α-promoter is shared with the adjacent *NBR2* gene and is bidirectional. In the present study, we screened a series of sporadic breast tumours to determine whether reductions in *BRCA1* mRNA levels can be attributed to mutations within the promoter region, and in the process also screened for new polymorphisms that could be associated with an increased risk of the disease.

*Received 20 May 1998*
*Revised 23 July 1998*
*Accepted 29 July 1998*

*Correspondence to:* A Catteau

### MATERIALS AND METHODS

### DNA samples

Blood and tumour DNA were extracted by standard methods. Details of the individuals studied using material derived from various centres in the UK are as follows:

*(i) Sporadic breast cancer patients*
Twenty paired tumour and blood DNA samples from patients with infiltrating adenocarcinoma were obtained from ICRF (Imperial Cancer Research Fund), Clinical Oncology Unit (Guy's Hospital). The average age of patients at diagnosis was 42.6 years (34–68); 18 patients were premenopausal and two were post-menopausal.

*(ii) Caucasian controls*
Forty-eight randomly selected (96 chromosomes) healthy, anonymous Caucasian individuals from the UK (25 women, 23 men; courtesy of Dr E. Maestrini, Oxford, UK) were analysed to determine the frequency of the G/C 1802 polymorphism in the general population.

*(iii) East Anglian series*
One hundred and forty-six East Anglian individuals with sporadic breast cancer or normal controls were analysed using material derived from CRC Human Cancer Genetics Research Group,

Addenbrooke's Hospital, Cambridge, UK. These subjects had previously been included in a large case–control study. Details concerning the recruitment and characteristics of these individuals have been provided elsewhere (Dunning et al, 1997).

### *(iv) Familial cases*

Four categories of patients ($n$ = 214) with a family history were screened for deletions in the promoter of *BRCA1* using genomic DNA extracted from peripheral blood lymphocytes. A family history was taken by a clinical geneticist or genetics nurse, and the diagnosis was confirmed in all probands and in affected relatives from whom information was available. The categories are defined as follows:

(1) patients with a family history of both breast and ovarian cancers ($n$ = 59);
(2) breast cancer patients with two affected first-degree relatives with at least one of the individuals diagnosed below the age of 45 years ($n$ = 50);
(3) breast cancer patients with one first-degree relative affected and at least one diagnosis before the age of 45 years ($n$ = 70);
(4) breast cancer patients with one second-degree relative affected and at least one diagnosis below the age of 45 years ($n$ = 35).

### Screening for mutations in the promoter region of the *BRCA1* gene by sequencing

Twenty sporadic breast cancer cases were screened for mutations by direct DNA sequencing. The promoter region of *BRCA1* was amplified by PCR using the tumour DNA as template. Two pairs of primer were used. The sequences of the primers were (forward/reverse, 5′–3′): TGGTATTGGATGTTCCTCTC/TTC-CAGTTCCTATCACGAGG and GCTCGCTGAGACTTCCTG/CCACAAGGTCCCATCCTCTC. Amplified products were purified with a Microspin™ S-400 column (Pharmacia Biotech, UK). The purified templates were sequenced in both directions with the PCR primers described above and the following additional primers (5′–3′): TCCAGGAAGTCTCAGCGAGCT and TAGGAACTG-GAATATGCCTTG. A dye terminator cycle sequencing kit (Applied Biosystem) was used and templates analysed on an ABI 377 DNA sequencer.

### Genotyping the C/G 1802 and Pro871Leu polymorphisms

The amplification refractory mutation system (ARMS) technique (Newton et al, 1989) was used to estimate the frequency of the C/G polymorphism in Caucasian population controls. The sequence of the reverse primer was (5′–3′) CACAAGGTCC-CATCCTCTC, while the sequence of the forward primer was (5′–3′) TGACAGATGGGTATTCTTTAAC and TGACAGAT-GGGTATTCTTTAAG for detecting the G and C alleles respectively. The amplification was for 30 cycles of denaturation at 94°C for 15 s, annealing at 55°C for 30 s and extension at 72°C for 1 min. An initial denaturation step of 5 min at 94°C and a final extension at 72°C for 5 min were used.

The linkage disequilibrium between the C/G 1802 and Pro871Leu polymorphisms was determined by analysis of a series of 146 East Anglian individuals previously genotyped for other polymorphisms including the Pro871Leu polymorphism using allele-specific oligonucleotide (ASO) hybridization (Dunning et al, 1997). The C/G polymorphism at nucleotide 1802 was detected by the ARMS technique as described above.

**Table 1** Linkage disequilibrium with allelic association between C/G 1802 and Pro871Leu in 146 individuals

| | PP | PL | LL |
|---|---|---|---|
| CC | 57 | 1 | |
| CG | 2 | 73 | |
| GG | | | 13 |
| Total | | | 146 |

Δ, 0.98; $\chi^2$, 279.

To screen for *BRCA1* promoter deletions in the familial breast cancer cases, ARMS was also used to determine the C/G 1802 genotype. All homozygotes (CC or GG) were analysed by direct DNA sequencing for the Pro871Leu polymorphism, which consists of a C to T change in exon 11 of *BRCA1* at nucleotide 35813 (accession no. L78833). The primers used were (forward/reverse, 5′–3′): GGGACTAATTCATGGTTGTTCC and TTTCTTTAAGGACCCAGAGTGG.

### Statistical analysis

The Hardy–Weinberg equilibrium in the populations studied was tested using a standard $\chi^2$ test. Association between genotypes at the polymorphic loci was also estimated by $\chi^2$ analysis. The strength of association was estimated by the correlation coefficient Δ (Chakravarti et al, 1984).

### RESULTS

### Screening for mutations in the *BRCA1* promoter in sporadic breast cancer and characterization of a novel polymorphism

Tumour DNA derived from 20 sporadic breast cancer patients was screened for mutations in the *BRCA1* promoter region. A 1197-bp fragment (position 1068–2264, accession no. U37574) encompassing both the α- and β-promoters (Xu et al, 1997) was analysed in detail by direct sequencing.

A C-to-G base change at nucleotide 1802, located ten bases downstream from an Sp1 site in the β-promoter, was detected. This C-to-G change was present in four subjects and was also detected in each of their peripheral blood DNA samples, suggesting that it represented a common variant rather than a disease-causing mutation. To confirm the presence of this polymorphism in the general population and to estimate its frequency, we genotyped 48 unrelated individuals (96 chromosomes) using the ARMS technique. The primers were carefully designed in order to prevent coamplification of the *BRCA1* pseudogene. The frequency of the C allele (published allele: Smith et al, 1996; Xu et al, 1997) was found to be 0.66 and the genotype distribution was in Hardy–Weinberg equilibrium ($\chi^2$ = 0.75; $P$ > 0.3). Apart from this polymorphism, no other base changes were identified.

If the C/G polymorphism itself affects, or is in linkage disequilibrium with, other genetic changes that cause variation of expression of *BRCA1*, it could ultimately be associated with breast cancer. In a recent case–control study, Dunning et al (1997) found that four common *BRCA1* polymorphisms are in strong linkage

 © Cancer Research Campaign 1999

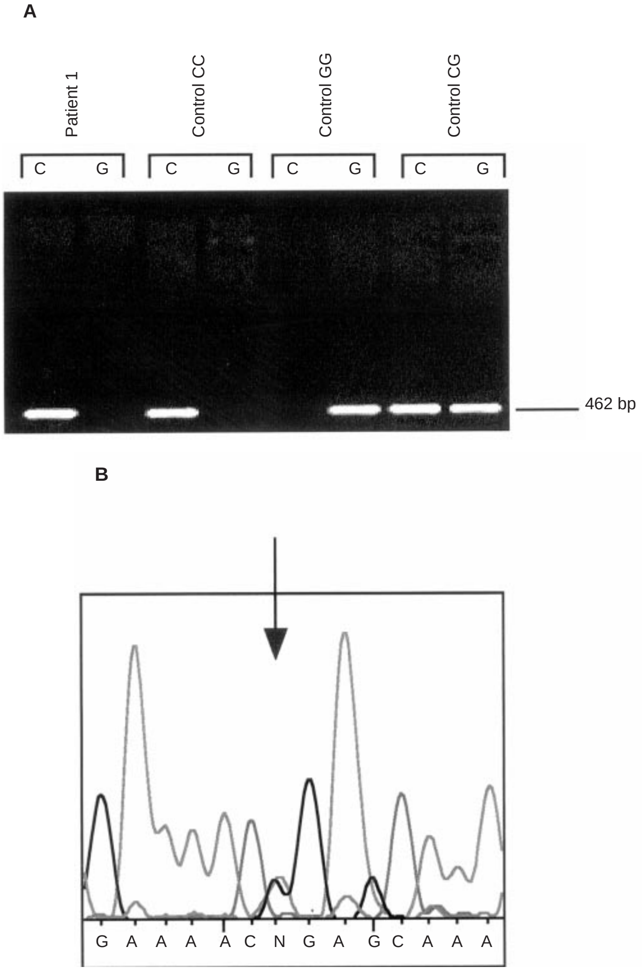

**Figure 1** Double genotyping of the C/G 1802 and Pro871Leu polymorphisms. Overall, 214 DNA samples extracted from blood obtained from familial breast cancer cases were analysed; 88 were found to be heterozygous for the C/G polymorphism (27, 18, 26 and 17 from categories 1, 2, 3 and 4 respectively – see Materials and methods for definitions), thereby excluding a deletion at this site. In 125 cases, homozygosity for both markers was observed: 24 cases were GG/LeuLeu (category 1, *n* = 6; 2, *n* = 2; 3, *n* = 9; 4, *n* = 7); 101 cases were CC/ProPro (category 1, *n* = 26; 2, *n* = 30; 3, *n* = 34; 4, *n* = 11). In the remaining case (patient 1), hemizygosity for the C/G marker and heterozygosity for the Pro871Leu polymorphism was observed as shown above, reflecting an underlying deletion in the *BRCA1* promoter. The C/G polymorphism was analysed by the ARMS technique (**A**) and the Pro871Leu polymorphism by automated sequencing (**B**)

disequilibrium and that none of the haplotypes is associated with a substantially increased risk of breast or ovarian cancer. Thus, we decided to investigate the allelic association between the newly identified C/G polymorphism and the Pro871Leu polymorphism analysed by Dunning et al (1997). One hundred and forty-six unrelated individuals from the original study (Dunning et al, 1997) were genotyped for the C/G polymorphism. Of the 292 alleles typed, three recombinant alleles were observed (1.03%, 95% CI 0.89–1.00), i.e. in 99% of alleles Pro-871 was associated with C and Leu-871 with G. Almost complete linkage disequilibrium was observed between the two polymorphic sites ($\Delta = 0.98$; $\chi^2 = 279$) (Table 1). No association was found between the Pro871Leu polymorphism and breast or ovarian cancer (Dunning et al, 1997); similarly, no significant risk of breast cancer could be attributed to the C/G 1802 polymorphism. The estimated relative risk to the G carriers is 1.06 (95% CI 0.87–1.30).

### Rapid screening for promoter deletions of *BRCA1* in familial breast cancer

As the recombination fraction is low between the C/G and Pro871Leu polymorphisms, they can therefore be used in combination as a marker for the detection of promoter deletions. According to this hypothesis, in individuals heterozygous for the Pro871Leu polymorphism, detection of apparent homozygosity at the C/G polymorphic site may be indicative of a deletion within the *BRCA1* promoter region, thus creating hemizygosity at this site. Screening of genomic DNA extracted from blood from a series of familial ($n = 214$) breast cancer cases was performed. The ARMS technique was used to characterize the C/G promoter polymorphism. The Pro871Leu polymorphism was analysed by sequencing in all cases found not to be heterozygous for the promoter marker. Some patients (88/214) were found to be heterozygous for the C/G polymorphism, thereby excluding a deletion in this region. Among the remaining cases, one individual was found to be homozygous for the C allele but heterozygous for the Pro871Leu polymorphism (see Figure 1), consistent with a deletion of the G allele in the *BRCA1* promoter. Southern analysis performed in this patient confirmed the presence of a deletion within the promoter region encompassing the C/G polymorphic site (Brown et al, manuscript in preparation).

### DISCUSSION

It is estimated that 45% of the breast cancer families are accounted for by defects in the *BRCA1* gene; however, in contrast to other tumour-suppressor genes, no somatic mutations have been identified within the coding region. Nevertheless, recent studies have shown that *BRCA1* transcripts are reduced in sporadic breast tumours, suggesting that *BRCA1* may be involved in both forms of the disease. The mechanism underlying the reduction in *BRCA1* levels is presently undetermined. However, by screening the promoter region in a series of 20 cases of sporadic breast cancer, the present study suggests that this is unlikely to be accounted for by mutations in either the α- or β-promoter of *BRCA1*. This would suggest that epigenetic mechanisms such as hypermethylation, deregulation of transcriptional activators and/or repressors binding the *BRCA1* locus or post-transcriptional processes could be involved. Indeed, there is evidence suggesting aberrant methylation within the regulatory region of *BRCA1* in sporadic breast tumors (Dobrovic and Simpfendorfer, 1997; Mancini et al, 1998; Catteau et al, in press).

Epidemiological data have suggested that the majority of breast cancer cases in the population might be accounted for by common variants that confer a modest risk of developing the disease rather than being due to highly penetrant genes (Ford et al, 1995). Examples of candidate variants leading to an increased risk of breast cancer are the *HRAS1* minisatellite locus (Krontiris et al, 1993), a polymorphism within the *NAT-2* gene (Ambrosone et al, 1995) and a polymorphism in the 5′ untranslated region of *CYP17* that creates an Sp1 site (Feigelson et al, 1997). In the process of examining the promoter region of *BRCA1*, we have detected a new polymorphism within the β-promoter consisting of a C-to-G base change at nucleotide 1802. We addressed whether it could represent a new biomarker for breast cancer risk. The base pair change occurs ten nucleotides downstream from an Sp1 site, one of the two transcription factor binding sites within the promoter region of *BRCA1* that are conserved between humans and mice. If the C-to-G base change affected the folding of the DNA and, in consequence, possibly the binding of transcription factors at the Sp1 site, a differential expression of *BRCA1* between both alleles could result, which could in turn lead to an interindividual risk of breast cancer. However, our preliminary data using functional in vitro assays suggest that the C/G polymorphism does not affect *BRCA1* expression. Furthermore, we demonstrated that there is close to complete allelic association between this polymorphism and the Pro871Leu polymorphism, which has itself been shown not to be associated with either breast or ovarian cancer in this population. Taken together, these results indicate that the C-to-G polymorphism is unlikely to make a significant contribution to either of these cancers.

Most of the *BRCA1* mutations detected in familial breast and ovarian cancers are small deletions, insertions or point mutations (Couch et al, 1996). However, recent results have highlighted the importance of large genomic deletions in *BRCA1* as a further mechanism leading to inactivation of the gene (Petrij-Bosch et al, 1997; Puget et al, 1997; Swensen et al, 1997). We and others have identified individuals with inferred putative regulatory mutations in *BRCA1* in familial cases with good evidence for linkage to *BRCA1* (Xu and Solomon, 1996), i.e. cases in which no mutations can be found by complete sequencing of the coding region yet only one allele is expressed. In such cases, if no mutation is detected in the promoter region, it is conceivable that the *BRCA1* gene is inactivated by a large genomic deletion which could be missed by PCR-based screening methods. As the *BRCA1* promoter region is complicated (Xu et al, 1995; Brown et al, 1996), containing two promoters and a pseudocopy, Southern analysis can be hindered by lack of informative probes and, moreover, by a paucity of DNA material. We have demonstrated that the combined genotyping of the C/G and the Pro871Leu polymorphisms is a powerful tool to screen for deletions in the *BRCA1* gene. Of 89 informative individuals with familial breast cancer, one case with a deletion involving the promoter region was successfully identified using this technique. While the use of this set of markers could miss deletions involving more distant sites, we suggest that the approach described here could be applied to other genomic regions of interest with the identification of further informative polymorphic markers.

In summary, we have identified a novel polymorphism in the promoter of *BRCA1*; while this does not predispose to breast cancer, we have found that it provides a valuable tool for rapid screening for germline mutations and suggest that it could also be used to determine loss of heterozygosity in sporadic cancer. We have demonstrated that deletion within the promoter region of *BRCA1* is an uncommon event in familial breast cancer. Furthermore, this study also suggests that mutations within the promoter are unlikely to account for the reduction in *BRCA1* levels that have been reported to be a frequent occurrence in sporadic disease.

### ACKNOWLEDGEMENTS

AC was supported by the ICRF (Imperial Cancer Research Fund) and Generation Trust, C-F X and MB by the UK MRC (Medical Research Council, Grant G6900577) and AMD by the CRC (Cancer Research Campaign). We are grateful to William Harris for technical assistance in obtaining tumour DNA samples, to Jill Greenman for DNA samples from the familial cases, to Dr E. Maestrini for DNA samples from Caucasian controls and to Dr David Grimwade for critical reading of the manuscript.

### REFERENCES

Ambrosone C, Freudenheim J, Marshall J, Graham S, Vena J, Brasure J, Michalek A, Laughlin R, Nemoto T and Shields P (1995) The association of polymorphic N-acetyltransferase (NAT 2) with breast cancer risk. *Ann New York Acad Sci* **768**: 250–252

Brown MA, Xu CF, Nicolai H, Griffiths B, Chambers JA, Black D and Solomon E (1996) The 5′ end of the *BRCA1* gene lies within a duplicated region of human-chromosome 17q21. *Oncogene* **12**: 2507–2513

Catteau A, Harris WH, Xu CF and Solomon E (1999) Methylation of the *BRCA1* promoter region in sporadic breast and ovarian cancer: correlation with disease characteristics. *Oncogene*

Chakravarti A, Buetow KH, Antonarakis SE, Waber PG, Boehm CD and Kazazian HH (1984) Nonuniform recombination within the human β-globin gene cluster. *Am J Hum Genet* **36**: 1239–1258

Couch FJ, Weber BL, Borresen AL, Brody L, Casey G, Devilee P, Fitzgerald M, Friend S, Gayther S, Goldgar D, Murphy P, Szabo C, Weber B, Wiseman R, Anderson T, Durocher F, Ganguly A, King MC, Lenoir G, Narod S, Olopade O, Plummer S, Ponder B, Serova O, Simard J, Stratton M and Warren B (1996) Mutations and polymorphisms in the familial early-onset breast-cancer (*BRCA1*) gene. *Hum Mutat* **8**: 8–18

Dobrovic A and Simpfendorfer D (1997) Methylation of the *BRCA1* gene in sporadic breast cancer. *Cancer Res* **57**: 3347–3350

Dunning AM, Chiano M, Smith NR, Dearden J, Gore M, Oakes S, Wilson C, Stratton M, Peto J, Easton D, Clayton D and Ponder BAJ (1997) Common *BRCA1* variants and susceptibility to breast and ovarian-cancer in the general population. *Hum Mol Genet* **6**: 285–289

Feigelson HS, Coetzee GA, Kolonel LN, Ross RK and Henderson BE (1997) A polymorphism in the CYP17 Gene increases the risk of breast cancer. *Cancer Res* **57**: 1063–1065

Ford D, Easton DF and Peto J (1995) Estimates of the gene-frequency of *BRCA1* and its contribution to breast and ovarian-cancer incidence. *Am J Hum Genet* **57**: 1457–1462

Futreal PA, Liu Q, Shattuck-Eidens D, Cochran C, Harshman K, Tavigian S, Bennett LM, Haugen-Strano A, Swensen J, Miki Y, Eddington K, McClure M, Frye C, Weaver-Feldhaus J, Ding W, Gholami Z, Soderkvist P, Terry L, Jhanwar S, Berchuck A, Iglehart JD, Marks J, Ballinger DG, Barrett JC, Skolnick MH, Kamb A and Wiseman R (1994) *BRCA1* mutations in primary breast and ovarian carcinomas. *Science* **266**: 120–122

Hosking L, Trowsdale J, Nicolai H, Solomon E, Foulkes W, Stamp G, Signer E and Jeffreys A (1995) A somatic *BRCA1* mutation in an ovarian tumor. *Nature Genet* **9**: 343–344

Krontiris TG, Devlin DB, Karp DD, Robert NJ and Risch N (1993) An association between the risk of cancer and mutations in HRAS1 minisatellite locus. *New Engl J Med* **329**: 517–523

Mancini DN, Rodenhiser DI, Ainsworth PJ, O'Malley FP, Singh SM, Xing W and Archer TK (1998) CpG methylation within the 5′ regulatory region of the *BRCA1* gene is tumor specific and includes a putative CREB binding site. *Oncogene* **16**: 1161–1169

Merajver SD, Pham TM, Caduff RF, Chen M, Poy EL, Cooney KA, Weber BL, Collins FS, Johnston C and Frank TS (1995) Somatic mutations in the *Brca1* gene in sporadic ovarian-tumors. *Nature Genet* **9**: 439–443

Miki Y, Swensen J, Shattuck-Eidens D, Futreal PA, Harshman K, Tavtigian S, Liu Q, Cochran C, Bennet LM, Ding W, Bell R, Rosenthal J, Hussey C, Tran T, McClure M, Frye C, Hattier T, Phelps R, Haugen-Strano A, Katcher H, Yakumo K, Gholami Z, Shaffer D, Sone S, Bayer S, Wray C, Bogden R, Dayananth P, Ward J, Tonin P, Narod S, Bristow PK, Norris FH, Helvering L, Morrison P, Rosteck P, Lai M, Barrett JC, Lewis C, Neuhausen S, Cannon-Albright L, Goldgar D, Wiseman R, Kamb A and Skolnick MH (1994) A strong candidate for the breast and ovarian cancer susceptibility gene *BRCA1*. *Science* **266**: 66–71

Newton CR, Graham A, Heptinstall LE, Powell SJ, Summers C, Kalsheker N, Smith JC and Markham AF (1989) Analysis of any point mutation in DNA. The amplification refractory mutation system (ARMS). *Nucleic Acios Res* **17**: 1503–1516

© Cancer Research Campaign 1999

Petrij-Bosch A, Peelen T, Van Vliet M, Van Eijk R, Olmer R, Drusedau M, Hogervorst FBL, Hageman S, Arts PJW, Ligtenberg MJL, Meijers-Heijboer H, Klijn JGM, Vasen HFA, Cornelisse CJ, Van't Veer LJ, Bakker E, Van Ommen G-JB and Devilee P (1997) *BRCA1* genomic deletions are major founder mutations in Dutch breast cancer patients. *Nature Genet* **17**: 341–345

Puget N, Torchard D, Serovasinilnikova OM, Lynch HT, Feunteun J, Lenoir GM and Mazoyer S (1997) A 1-kb alu-mediated germ-line deletion removing *BRCA1* exon-17. *Cancer Res* **57**: 828–831

Smith TM, Lee MK, Szabo CI, Jerome N, McEuen M, Taylor M, Hood L and King MC (1996) Complete genomic sequence and analysis of 117 kb of human DNA containing the gene *BRCA1*. *Genome Res* **6**: 1029–1049

Swensen J, Hohhman M, Skolnick MH and Neuhausen SL (1997) Identification of a 14 kb deletion involving the promoter region of *BRCA1* in a breast cancer family. *Hum Mol Genet* **6**: 1513–1517

Thompson ME, Jensen RA, Obermiller PS, Page DL and Holt JT (1995) Decreased expression of *BRCA1* accelerates growth and is often present during sporadic breast-cancer progression. *Nature Genet* **9**: 444–450

Xu C-F, Brown MA, Chambers JA, Griffiths B, Nicolai H and Solomon E (1995) Distinct transcription start sites generate two forms of *BRCA1* mRNA. *Hum Mol Genet* **4**: 2259–2264

Xu C-F and Solomon E (1996) Mutations of the *BRCA1* gene in human cancer. *Semin Cancer Biol* **7**: 33–40

Xu C-F, Chambers JA and Solomon E (1997) Complex regulation of the *BRCA1* gene. *J Biol Chem* **272**: 20994–20997

*© Cancer Research Campaign 1999*
